# Multi-angle quantum approximate optimization algorithm

**DOI:** 10.1038/s41598-022-10555-8

**Published:** 2022-04-26

**Authors:** Rebekah Herrman, Phillip C. Lotshaw, James Ostrowski, Travis S. Humble, George Siopsis

**Affiliations:** 1 Department of Industrial and Systems Engineering, University of Tennessee at Knoxville, Knoxville, TN 37996 USA; 2 Quantum Computing Institute, Oak Ridge National Laboratory, Oak Ridge, TN 37830 USA; 3 Department of Physics and Astronomy, University of Tennessee at Knoxville, Knoxville, TN 37996 USA

**Keywords:** Quantum information, Computational science

## Abstract

The quantum approximate optimization algorithm (QAOA) generates an approximate solution to combinatorial optimization problems using a variational ansatz circuit defined by parameterized layers of quantum evolution. In theory, the approximation improves with increasing ansatz depth but gate noise and circuit complexity undermine performance in practice. Here, we investigate a multi-angle ansatz for QAOA that reduces circuit depth and improves the approximation ratio by increasing the number of classical parameters. Even though the number of parameters increases, our results indicate that good parameters can be found in polynomial time for a test dataset we consider. This new ansatz gives a 33% increase in the approximation ratio for an infinite family of MaxCut instances over QAOA. The optimal performance is lower bounded by the conventional ansatz, and we present empirical results for graphs on eight vertices that one layer of the multi-angle anstaz is comparable to three layers of the traditional ansatz on MaxCut problems. Similarly, multi-angle QAOA yields a higher approximation ratio than QAOA at the same depth on a collection of MaxCut instances on fifty and one-hundred vertex graphs. Many of the optimized parameters are found to be zero, so their associated gates can be removed from the circuit, further decreasing the circuit depth. These results indicate that multi-angle QAOA requires shallower circuits to solve problems than QAOA, making it more viable for near-term intermediate-scale quantum devices.

## Introduction

Among several quantum algorithms implemented on noisy intermediate-scale quantum (NISQ) devices^1–12^, the quantum approximate optimization algorithm (QAOA) offers an opportunity to approximately solve combinatorial optimization problems such as MaxCut, Max Independent Set, and Max k-cover^13–22^. QAOA tunes a set of classical parameters to optimize the cost function expectation value for a quantum state prepared by well-defined sequence of operators acting on a known initial state. Variations to the original algorithm include alternative operators and initial states^23–30^ while purely classical aspects such as the parameter optimization and problem structure have been tested as well^31–36^. However, an outstanding concern is that practical implementations of QAOA require large numbers of qubits and deep circuits^37^. For example, a recent study has developed a systematic set of parameters that are argued to require $p=30$ layers of QAOA to reach performance comparable to the conventional Goemans–Williamson algorithm on MaxCut^36^, while another study has argued that hundreds of qubits or more are needed to compete with conventional solvers in time-to-solution^38^. Noise grows rapidly with circuit depth and affects the fidelity of the prepared quantum state so the performance that can be achieved from near-term quantum computers at these depths is questionable^39–49^.

One approach to reduce the circuit depth of QAOA is to increase the number of classical parameters introduced in each layer, a variation that we term multi-angle QAOA (ma-QAOA). This approach was originally briefly introduced in^50^. Increasing the number of classical parameters allows for finer-grain control over the optimization of the cost function and the approximation ratio, which measures optimality relative to the known best solution. While introducing more classical parameters can lead to a more challenging optimization, a corresponding reduction in circuit depth preserves the critical resource of the quantum state. In addition, finding the absolute optimal angles is not necessary in order to see an improvement over QAOA.

Here, we quantify the advantages of using multiple parameters for each layer of QAOA. First, we prove that the approximation ratio converges to one as the number of iterations of ma-QAOA tends to infinity, a property that ensures the optimal solution is the most likely. We next demonstrate that one iteration of ma-QAOA gives an approximation ratio that is at least that of the approximation ratio after one iteration of QAOA. This shows that ma-QAOA performs at least as well as QAOA. We also show that ma-QAOA used to solve the MaxCut problem on star graphs achieves an approximation ratio of one after one iteration, while single-iteration QAOA tends to an approximation ratio of 0.75 as the number of vertices goes to infinity. This result gives a concrete example where ma-QAOA gives a strictly larger approximation ratio than QAOA. We simulate solving MaxCut using ma-QAOA and QAOA on all connected, non-isomorphic eight vertex graphs and compare the performance of the two ansatzes. In doing so, we find that the average approximation ratio for ma-QAOA after one iteration is larger than the average approximation ratio of QAOA after three iterations. In looking at larger, fifty and one-hundred vertex graphs, we see that ma-QAOA retains its advantage over QAOA, giving approximation ratios that are on average six percentage points higher after the first iteration.

## Results

### Multi-angle quantum approximate optimization algorithm

We develop the multi-angle QAOA beginning with the standard formulation of the quantum approximate optimization algorithm (QAOA). The QAOA relies on a combination of classical parameter optimization and applying cost and mixing operators to a quantum state in order to approximately solve combinatorial optimization (CO) problems^13^. CO problems are defined by an objective function, *C*(*z*), where *z* is a bit string of length *n*. Often, *C*(*z*) is the sum over a collection of clauses,

$$\begin{aligned} C(z) = \sum _{a} C_a(z). \end{aligned}$$

When solving these problems with QAOA, *C*(*z*) is encoded into a matrix *C* with eigenvalues given by the classical cost values

1 $$\begin{aligned} C\vert z \rangle = C(z) \vert z \rangle . \end{aligned}$$

QAOA requires two operators,

$$\begin{aligned} U(\gamma , C) = e^{-i\gamma C} \end{aligned}$$

and

$$\begin{aligned} U(\beta , B) = e^{-i\beta B} \end{aligned}$$

which have real-valued angle inputs $\gamma \in [0,2\pi )$ and $\beta \in [0, \pi )$. *B* drives transitions between computational basis states and is typically

$$\begin{aligned} B = \sum _{v} B_v \end{aligned}$$

where $B_v = \sigma _v^x$ is the Pauli-x operator acting on qubit *v* in the quantum system. The two operators are applied to an initial state,

$$\begin{aligned} {\left| s \right\rangle }=\frac{1}{\sqrt{2^n}} \sum _{z} {\left| z \right\rangle }. \end{aligned}$$

Here the sum is over the computational basis ${\left| z \right\rangle }$. The QAOA ansatz operator applied *p* times to ${\left| s \right\rangle }$ is denoted *p*-QAOA. The state for *p*-QAOA is

$$\begin{aligned} {\left| \gamma ,\beta \right\rangle }=U(\beta _p, B)U(\gamma _p,C)...U(\beta _1, B)U(\gamma _1,C){\left| s \right\rangle }. \end{aligned}$$

Since *C* and *B* are sums of matrices, we may write

$$\begin{aligned} U(\gamma , C) = e^{-i\gamma \sum _{a}C_a } \end{aligned}$$

and

$$\begin{aligned} U(\beta , B) = e^{-i\beta \sum _{v}B_{v}}. \end{aligned}$$

Instead of focusing on minimizing the classical optimization efforts in QAOA, QAOA can be modified such that it requires more classical parameters^50^. The new classical parameters are introduced to QAOA by allowing each summand of the cost and mixing operators to have its own angle instead of a single angle for the cost operator and a second angle for the mixing operator. In this modification,

$$\begin{aligned} U(\vec {\gamma _l},C) = e^{-i \sum _{a}\gamma _{l,a}C_a } = \prod _{a}e^{-i\gamma _{l,a} C_a } \end{aligned}$$

and

$$\begin{aligned} U(\vec {\beta _l}, B) = e^{-i \sum _{v}\beta _{l,v}B_{v} } = \prod _{v}e^{-i\beta _{l,v} B_v } \end{aligned}$$

where $\vec {\gamma _l} = (\gamma _{l,a_1}, \gamma _{l,a_2}, ... )$ and $\vec {\beta _l} = (\beta _{l,v_1}, \beta _{l,v_2}, ... )$. Here, *l* denotes the layer, $a_i$ denotes a specific clause, and $v_j$ refers to a specific qubit. We call this modification multi-angle QAOA and abbreviate it ma-QAOA. Similarly to QAOA, when the operators for ma-QAOA are applied *p* times to the initial state, we call this *p*-ma-QAOA.

The performance of the algorithm is typically characterized by the approximation ratio, denoted A.R.,

2 $$\begin{aligned} \mathrm {A.R.} = \frac{\langle C \rangle }{C_\mathrm {max}} \end{aligned}$$

which compares the expectation value of the cost operator $\langle C \rangle$ to the optimal solution value $C_\mathrm {max}$ . We will write $\langle C\rangle = \langle C\rangle _p$ for *p*-QAOA and $\langle C \rangle = \langle C\rangle _p^\mathrm {ma}$ for *p*-ma-QAOA.

### Convergence of ma-QAOA

For QAOA, the expected value of *C* after *p* iterations is $\langle C \rangle _p = {\left\langle \gamma , \beta \right| }C{\left| \gamma , \beta \right\rangle }$. Let $M_p$ be the maximum of $\langle C \rangle _p$ over all angles. Then, $M_p \ge M_{p-1}$. Farhi, Goldstone, and Gutmann showed that $M_p$ tends to the maximum of the objective function, $C_\mathrm {max}$, for the CO problem being solved as *p* tends to infinity^13^.

We similarly define the expected value of *C* after *p* iterations of ma-QAOA as $\langle C \rangle _p^\mathrm {ma} = {\left\langle \vec {\gamma }_{\mathrm {ma}}, \vec {\beta }_{\mathrm {ma}} \right| }C{\left| \vec {\gamma }_{\mathrm {ma}}, \vec {\beta }_{\mathrm {ma}} \right\rangle }$ where $\vec {\gamma }_{\mathrm {ma}} = (\vec \gamma _1, \vec \gamma _2,...\vec \gamma _p)$ and $\vec {\beta }_{\mathrm {ma}} = (\vec \beta _1, \vec \beta _2,...\vec \beta _p)$. We also define $M_p^\mathrm {ma}$ to be the maximum of $\langle C \rangle _p^\mathrm {ma}$ over all angles. Clearly, $M_p^\mathrm {ma} \ge M_p$ because QAOA is the special case of ma-QAOA where $\beta _{p,u} = \beta _{p,v}$ for all $u \ne v$ and $\gamma _{p,a_i} = \gamma _{p,a_j}$ for edges $a_i \ne a_j$.

In order to show ma-QAOA gives the optimal solution to a combinatorial optimization problem, we must show $\langle C \rangle _p^\mathrm {ma}$ converges to $C_\mathrm {max}$ as *p* tends to infinity. Convergence is the first main result of this work.

#### Theorem 2.1

*The multi-angle quantum approximate optimization algorithm converges to the optimal solution of a combinatorial optimization problem as* $p \rightarrow \infty$.

The proof of convergence is given in section “Methods”.

### MaxCut problem and performance on star graphs

In graph theory, a graph $G = (V,E)$ consists of a collection of vertices, *V*, and edges, *E*, which are pairs of vertices. MaxCut is a CO problem defined with respect to a graph. For QAOA, each qubit corresponds to a vertex in *G* and the cost operator is^13^

$$\begin{aligned} C = \sum _{ij \in E} \frac{1}{2}(-\sigma _i^z\sigma _j^z + 1). \end{aligned}$$

The goal of the problem is partition the vertices into two sets such that the number of edges with endpoints in each set is maximized.

**Figure 1:**
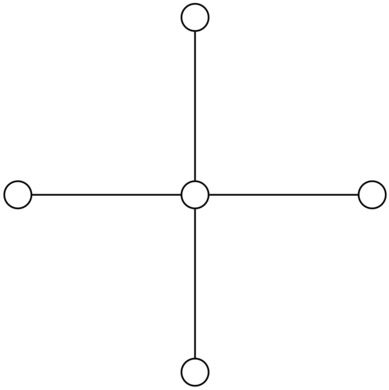
The star graph on five vertices.

A star graph on *n* vertices is a graph that consists of one vertex of degree $n-1$, called the center. All other vertices of the graph have degree one, meaning each vertex is connected to the center and only the center. An example can be seen in Fig. 1. All stars are trees, and are thus bipartite, so the optimal MaxCut solution includes all edges of the graph. In order to show ma-QAOA outperforms QAOA when solving MaxCut on star graphs, we show that $\langle C \rangle _1^\mathrm {ma} = 1$ and $\langle C \rangle _1$ tends to 0.75 as *n* tends to infinity. The proof is found in section “Methods”.

### Computational results

In order to test how ma-QAOA performs, we simulated the algorithm on a collection of one-hundred triangle-free 3-regular graphs with fifty vertices and one-hundred triangle-free 3-regular graphs with 100 vertices and compared the approximation ratios calculated with ma-QAOA to those of 1-QAOA. We also performed the same calculations with fifty modified $G_{n,p}$ random graphs with fifty and one-hundred vertices each; approximation ratio results for all large graphs are summarized in Table 1. In the $G_{n,p}$ model, *n* sets the number of vertices, and *p* is the probability that an edge exists. In particular, we examined $G_{50, 0.08}$ and $G_{100, 0.035}$ in order to create random graphs that have average degree approximately three. After randomly generating the graphs, triangles were removed by randomly removing edges from each triangle. For these sets of triangle-free graphs we can compute $\langle C \rangle _1^\mathrm {ma}$ for large *n* using the analytical result of Theorem 4.1. Table 1 shows the average approximation ratios for each collection of graphs with ma-QAOA and 1-QAOA, as well as the changes in the approximation ratio and percent change in the approximation ratio gap. This approximation ratio gap is the percent difference between one minus the approximation ratio for 1-QAOA and one minus the approximation ratio for ma-QAOA. The ma-QAOA has a higher average approximation ratio and gives a significant percent increase in approximation ratio gap for each collection of graphs. These simulations only compare 1-QAOA to 1-ma-QAOA, however, the next set of computational results compares 1-ma-QAOA to *p*-QAOA for $p \le 3$ on all connected, non-isomorphic graphs.

**Figure 2:**
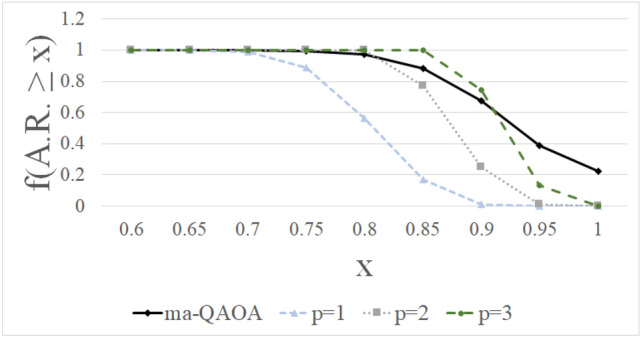
The fraction of non-isomorphic, eight-vertex graphs with approximation ratios at least X ($\mathrm {f(A.R.} \ge \mathrm {X}$)) for 1-ma-QAOA and *p*-QAOA. The lines are included in order to outline the shape of each distribution.

In previous work, we determined $\langle C \rangle _1$, $\langle C \rangle _2$, and $\langle C \rangle _3$ for all connected, non-isomorphic eight vertex graphs and compiled them into an online data set^35,51^. For this work, we calculated the angles that maximize $\langle C \rangle _1^{\mathrm {ma}}$ and compared $\langle C \rangle _p$ to $\langle C \rangle ^{\mathrm {ma}}_1$. On average, the performance of ma-QAOA is comparable to 3-QAOA on these graphs. Table 2 shows that ma-QAOA has a higher average approximation ratio than 1-QAOA and 2-QAOA on all eight vertex graphs. However, the average approximation ratio for one iteration of ma-QAOA is larger than the average approximation ratio for 3-QAOA.

**Table 1:** The average approximation ratio (A.R.) for a collection of one-hundred 3-regular graphs with fifty vertices, one-hundred 3-regular graphs with 100 vertices, fifty modified $G_{50,0.08}$ random graphs, and fifty modified $G_{100,0.035}$ random graphs.

Graph type	Average A.R. for 1-QAOA	Average A.R. for ma-QAOA	Change in A.R.	Percent change in gap (1-AR)
50 vertex 3-regular	0.7617	0.8123	0.0506	21.26%
100 vertex 3-regular	0.7562	0.8000	0.0438	17.98%
Modified $G_{50,0.08}$	0.7554	0.8156	0.0602	24.65%
Modified $G_{100,0.035}$	0.7497	0.8098	0.0602	24.04%

**Table 2:** The average approximation ratio for all connected, non-isomorphic graphs eight vertices.

QAOA type	Average approximation ratio for all eight vertex graphs
ma-QAOA	0.9257
1-QAOA	0.8061
2-QAOA	0.8767
3-QAOA	0.9192

Figure 2 shows how the distribution of approximation ratios for ma-QAOA compares to the approximation ratios for up to three iterations of QAOA for all connected, non-isomorphic eight vertex graphs. The percentage of graphs with approximation ratio at least 0.95 is significantly higher with ma-QAOA than up to three levels of QAOA. The fraction of graphs with approximation ratio at least 0.85 and 0.9 is higher for 3-QAOA than ma-QAOA, however significantly more graphs have an approximation ratio of at least 0.95 with ma-QAOA.

### Measurement reliability

We quantify the number of measurements to obtain a reliable result from ma-QAOA and QAOA using a simple noise model with Kraus-operator error channels acting after each unitary operator in the ansatz. On fully connected hardware, the numbers of one-qubit unitary operators and two-qubit unitary operators per iteration of QAOA for MaxCut equal the numbers of vertices *n* and edges *m* in the graph, respectively. On connected $n=8$ vertex graphs, $7 \le m \le 28$. Following these unitary and channel operators, the circuit produces a final state $\rho = F \rho _\mathrm {ideal} + (1-F)\rho _\mathrm {noise}$, where *F* is the probability associated with the ideal noiseless evolution component $\rho _\mathrm {ideal}$^52^. Assuming error rates of $\epsilon _n$ and $\epsilon _m$ for each one- and two-qubit unitary respectively, $F = (1-\epsilon _{n})^{np}(1-\epsilon _{m})^{mp}$.

A measurement projects $\rho$ onto a basis state $\vert z \rangle$ and the total set of measurement probabilities is described by $\rho '=\sum _z \Pi _z \rho \Pi _z$, with $\Pi _z = \vert z \rangle \langle z \vert$. The expected number of measurements to sample a result $\vert z \rangle$ from the ideal distribution is 1/*F* in the worst-case^48^, when $\mathrm {Tr}\rho _\mathrm {ideal}'\rho _\mathrm {noise}'=0$; the number of measurements can decrease depending on the specific state and noise process, but to keep the discussion general we take the expected number of measurements as 1/*F* . We compute *F* using the average numbers of edges $\langle m \rangle$ for graphs in our datasets, for example $\langle m\rangle =14.4$ at $n=8$, but note each specific graph has an integer number of edges. Assuming $p=1$, $n=8$, $\langle m \rangle =14.4$, and an error rate of 1% for each unitary operator, the expected number of measurements to obtain a sample from the noiseless distribution is 1.25.

We find that parameter optimization with ma-QAOA yields angles of zero for a subset of the edge and vertex unitary operators and we use this in the calculation of *F*. Since $\exp (-i \gamma _{p,a}C_{a})={\mathbb {I}}=\exp (-i\beta _{p,v}B_v)$ when $\gamma _{p,a}=0$ and $\beta _{p,v} = 0$, all unitary operators with an angle of zero may be excluded from the optimized circuit. This decreases the exponent of the first and second terms in *F* by the number of vertex and edge operators that have zero angles, respectively, and thus reduces the amount of noise in ma-QAOA relative to QAOA. Table 3 gives the percent of zero angles, rounded to three decimal places, for each collection of graphs that were studied.

Table 4 shows the ratio of the expected number of measurements needed to sample from the noiseless distribution for *p*-QAOA relative to ma-QAOA for each collection of graphs with varying values of $\epsilon _{\langle m \rangle }$, using the average reduction in gates for ma-QAOA from Table 3. Note that if the $\epsilon _{\langle m \rangle } =0.05$, the number of samples increases rapidly with *p*.

From the performance bound of Theorem 2.1, ma-QAOA will never need more layers than standard QAOA to reach a given approximation ratio. Whenever standard QAOA requires more layers than ma-QAOA, the additional noise from these layers will lead to an increase in the number of samples that are needed according to our model. Since one iteration of ma-QAOA is comparable to three iterations of QAOA on eight vertex graphs, if the trend holds for larger graphs, ma-QAOA has the potential to require significantly fewer samples than QAOA.

**Table 3:** The percent of $\beta _v$ and $\gamma _a$, rounded to three decimal places, that are zero when optimizing ma-QAOA on the family of graphs found in the first column.

*n*	Percent of *v* with $\beta _v = 0$	Percent of *a* with $\gamma _a = 0$
8	15.030	25.449
50 (3-reg.)	13.000	18.147
50 (E.R.)	11.440	14.381
100 (3-reg.)	14.690	19.973
100 (E.R.)	12.900	16.541

**Table 4:** The ratio of the expected number of measurements to obtain a sample from the noiseless distribution for *p*-QAOA relative to 1-ma-QAOA on an *n* vertex graph, assuming an average number of edges $\langle m \rangle$ for graphs in the datasets.

*n*	$\langle m \rangle$	$\epsilon _n =\epsilon _{\langle m \rangle } = 0.01$			$\epsilon _n = 0.01, \epsilon _{\langle m \rangle } = 0.05$		
		$p=1$	$p=2$	$p=3$	$p=1$	$p=2$	$p=3$
8	14.4	1.05	1.32	1.65	1.22	2.77	6.28
50 (3-reg.)	75	1.22	4.30	15.10	2.15	166.16	$1\times 10^4$
50 (E.R.)	87.2	1.20	4.77	18.94	2.02	291.78	$4\times 10^4$
100 (3-reg.)	150	1.57	19.32	238.39	5.39	$3\times 10^4$	$2\times 10^8$
100 (E.R.)	167.34	1.50	22.08	324.26	4.71	$7\times 10^4$	$1\times 10^9$

### Computing angles

With a larger number of variables to optimize, the ma-QAOA method requires more classical effort to find angles that optimize the approximation ratio. However, it is not necessary to identify exact optimal angles, only to find angles that are better than QAOA angles.

**Figure 3:**
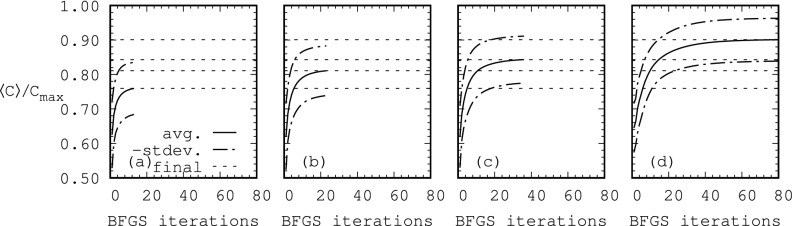
Typical behavior of the BFGS search algorithm for 100 random eight vertex graphs each with 100 random seeds in BFGS, for regular QAOA at (**a**) $p=1$, (**b**) $p=2$, (**c**) $p=3$ and for (**d**) ma-QAOA at $p=1$. Solid curves show averages over graphs and seeds, dashed-dotted lines show the average ± standard deviation and dotted lines show the final values. Each curve terminates at the average final iteration of the BFGS algorithm for the dataset.

We used the Broyden-Fletcher-Goldfarb-Shanno (BFGS) algorithm to compute angles for the 8-vertex graphs; details can be found in “Methods” section. Figure 3 shows how the approximation ratio improves on average across all iterations of BFGS for each ansatz studied for a random sample of eight vertex graphs. Note that after approximately ten iterations, ma-QAOA tends to achieve a higher approximation ratio than any of the *p*-QAOA. We do note that the time required to perform each iteration of BFGS is slower for ma-QAOA, as the number of gradient components is linearly dependent on the number of variables being optimized.

### Scaling

We assess the scalability of ma-QAOA using computed optimized $\langle C \rangle$ for sets of triangle-free Erdős-Rényi and 3-regular graphs with $n=50$ and $n=100$ vertices. The computational details are given in section “Methods”. We compare the run times for typical graph optimizations to assess how the ma-QAOA parameter optimization time increases with graph size.

For the Erdős-Rényi graphs, the time for a single optimization for $n=50$ was 0.10 seconds, for $n=100$ it was 0.46 seconds. We attribute the difference primarily to the scaling in the calculation of the gradient, which is the most expensive calculation in the optimization. Our approach computes each of the $n+m$ derivatives $\partial \langle C_{p,uv}\rangle ^\mathrm {ma}/\partial \beta _{p,w}$ and $\partial \langle C_{p,uv}\rangle ^\mathrm {ma}/\partial \gamma _{p,jk}$ for each of the *m* terms $\langle C_{p,uv}\rangle ^\mathrm {ma}$ in the cost function, giving a total number of terms $\sim (n+m)m$. The time to compute each term will vary with the degree of the graph, as this determines the number of cosine terms in Theorem 4.1; however, for our graphs the degree is approximately constant hence can be neglected in the scaling. For our graphs $m \sim n$ on average, so the overall scaling is $\sim n^2$, which is consistent with the $\approx 4\times$ increase in time when *n* is doubled from $n=50$ to $n=100$. We attribute the remainder of the time difference to variations in the number of iterations as *n* and *m* increase.

It is interesting to consider scaling of the optimization time with the number of vertices *n* for instances beyond the current dataset. For a gradient-based optimization this requires computing $\partial \langle C^\mathrm {ma}\rangle /\partial \theta =\sum _a \partial \langle C_a \rangle /\partial \theta$ for each parameter $\theta$, for each step in the optimization. For MaxCut and a variety of other problems^53^, the number of clauses $C_a$ is poly(*n*), and so there are poly(*n*) parameters and poly(*n*) partial derivatives $\partial \langle C_a \rangle /\partial \theta$ in the gradient. There are situations in which the time to compute each $\partial \langle C_a \rangle /\partial \theta$ is independent of *n*, specifically, when *p* and the graph structure are fixed such that each partial derivative can be computed using *n*-independent “sub-graphs”^13^. Then we need to compute poly(*n*) terms with fixed compute time per term, so the overall time to compute the gradient scales as poly(*n*). The gradient based optimization approach BFGS exhibits super-linear convergence on a variety of practical problems^54^, which supports the idea that the number of steps will not scale problematically with *n*. Perhaps counterintuitively, a recent investigation of variational quantum algorithms suggests that algorithms with more parameters have fewer local optima and achieve better convergence to global optima^55^, suggesting ma-QAOA may require fewer BFGS step to optimize than standard QAOA.

## Discussion

We have shown that multi-angle QAOA converges to an optimal solution, and furthermore that $\langle C \rangle _1^\mathrm {ma} \ge \langle C \rangle _1$, as QAOA is a special case of ma-QAOA. Additionally, the analysis of star graphs shows that there is a family of graphs that always gives larger $\langle C \rangle$ for MaxCut when solved with ma-QAOA than when solved with QAOA. We find significant increases in the approximation ratio in numerical optimizations for large triangle-free graphs and over the set of all non-isomorphic graphs with eight vertices, hence fewer layers are required to reach the same performance as QAOA. We also show that optimized rotation angles are often zero in ma-QAOA and this reduces the number of unitary operators per layer relative to QAOA. In the presence of noise, the reduction in number of layers and in the number of unitary operators per layer can significantly decrease the expected number of measurements needed to sample a result $\vert z \rangle$ in the distribution of the noiseless state. This could be a significant advantage for computations on noisy quantum hardware.

Interestingly, some graphs do not have a significantly higher $\langle C \rangle$ when solving MaxCut with ma-QAOA versus QAOA. It would be useful to characterize for which graphs the increase in $\langle C \rangle$ from QAOA to ma-QAOA is insignificant. This would help determine the appropriate ansatz to use in order to solve MaxCut on the graph.

One drawback to ma-QAOA is that the number of classically optimized parameters is $n+m$ per layer, where *n* is the number of vertices of *G* and *m* is the number of edges. An argument can be made that if *x* parameters are required to optimize one iteration of ma-QAOA, the results should be compared to QAOA with the same number of parameters. This approach would require $p \approx \frac{x}{2}$ iterations of QAOA, which may not be feasible on current hardware as a large number of layers will accumulate considerable noise. From this perspective, it is advantageous to incorporate additional parameters into a small number of circuit layers. It could be interesting to consider the comparison with the same numbers of parameters from a theoretical perspective, but it is beyond our scope here.

From a practical standpoint, one way to solve optimal ma-QAOA angles would be to calculate $\beta$ and $\gamma$ that optimize QAOA. We can use those angles as the initial point of a BFGS search for the optimal $\beta _{p,v}$ and $\gamma _{p, a_i}$ for all vertices *v* and edges $a_i$. Overall, however, the results seem to indicate that good parameters can be found in polynomial time. As many combinatorial optimization problems, like MaxCut, are NP-Hard, any polynomially-bounded effort that improves performance is likely to improve performance at large scale.

## Methods

### Proof of convergence

#### Proof

Recall that QAOA converges to the optimal solution for a combinatorial optimization problem, which is the maximum over the objective function^13^. Thus, in order to show convergence of ma-QAOA, we need only bound ma-QAOA from below by the value of QAOA. However, it is clear that the optimal expected value of the cost function for ma-QAOA can be no lower than that of QAOA, since QAOA is a special case of ma-QAOA when all $\gamma _{p,ij} = \gamma _{p,kl}$ and all $\beta _{p,a} = \beta _{p,b}$ for all edges *ij*, *kl* and all vertices *a*, *b*. $\square$

### Formula for $\langle C \rangle$

In order to prove that $\langle C \rangle _1^\mathrm {ma} = 1$ for MaxCut on star graphs, we derive a formula that calculates $\langle C \rangle _1^\mathrm {ma}$ for MaxCut on triangle-free graphs.

#### Theorem 4.1

Let $\beta _{p,u}' = 2\beta _{p,u}$ and $\beta _{p,v}' = 2\beta _{p,v}$ The expected value of *C* after one iteration of ma-QAOA applied to MaxCut for triangle-free graphs *G* is

$$\begin{aligned}{\left\langle \vec {\gamma _1}\vec {\beta _1} \right| } C_{uv} {\left| \vec {\gamma _1}\vec {\beta _1} \right\rangle } = \frac{1}{2}+ \frac{1}{2}\sin {\gamma _{1,uv}}(\cos {\beta _{1,v}'}\sin {\beta _{1,u}'}\prod _w \cos {\gamma _{1,uw}}+ \cos {\beta _{1,u}'}\sin {\beta _{1,v}'} \prod _{x}\cos {\gamma _{1,vx}}) \end{aligned}$$

where $$w \in Nbhd(u)\setminus v$$ and $$x \in Nbhd(v)\setminus u$$.

The neighborhood of a vertex *x*, denoted *Nbhd*(*x*), is the set of vertices *y* such that $xy \in E(G)$.

#### Proof

The proof of this result relies on the Pauli-solver algorithm, which is explained in detail in^56^. The proof of the result is virtually identical to that for QAOA on triangle-free graphs, but we include the proof here for completeness.

Consider edge *uv* and consider acting on $C_{uv}= (1/2)({\mathbb {I}}-Z_uZ_v)$ by conjugation of the mixing operator, $\prod _{i \in V}e^{-i \beta _{1,i} B_i},$ followed by conjugation of the phase operator, $\prod _{uv \in E} e^{-i \gamma _{1,uv} C_{uv}}$. We have that

3 $$\begin{aligned} \begin{aligned} \prod _{i \in V}e^{i \beta _{1,i} B_i}Z_uZ_v\prod _{i \in V}e^{-i \beta _{1,i} B_i}&= e^{2i\beta _{1,u} X_u}e^{2i\beta _{1,v} X_v}Z_uZ_v \\&= \cos {2\beta _{1,u}}\cos {2\beta _{1,v}}Z_uZ_v+ \cos {2\beta _{1,v}}\sin {2\beta _{1,u}}Y_vZ_u \\&\quad +\cos {2\beta _{1,u}}\sin {2\beta _{1,v}}Z_vY_u+\sin {2\beta _{1,u}}\sin {2\beta _{1,v}}Y_uY_v. \end{aligned} \end{aligned}$$

Note that the first term commutes with $\prod _{uv \in E} e^{-i \gamma _{1,uv} C_{uv}}$, so does not contribute to the expected value. Let $V_u$ be the neighborhood of *u* in *V*(*G*). Conjugating the third term of Eqn. (3) by $\prod _{uv \in E} e^{-i \gamma _{1,uv} C_{uv}}$, we get

$$\begin{aligned} {\left\langle s \right| }\Upsilon ^\dag Y_uZ_v\Upsilon {\left| s \right\rangle }&= {\left\langle s \right| }e^{2i \gamma _{1,uv} C_{uv}}e^{2i \sum _{a \in V_u\setminus {v}} \gamma _{1,ua} C_{ua} }Y_uZ_v{\left| s \right\rangle } \\&= {\left\langle s \right| }e^{-i \gamma _{1,uv} Z_uZ_v}e^{-i \sum _{a \in V_u \setminus {v}} \gamma _{1,ua} Z_uZ_a }Y_uZ_v{\left| s \right\rangle } \\&= {\left\langle s \right| }({\mathbb {I}}\cos {\gamma _{1,uv}}-i\sin {\gamma _{1,uv}})Z_uZ_v \\&\quad \prod _{a \in V_u}({\mathbb {I}}\cos {\gamma _{1,ua}}-i\sin {\gamma _{1,ua}}Z_uZ_a)Y_uZ_v{\left| s \right\rangle } \\&= -\sin {\gamma _{1,uv}}\prod _{a \in V_u}\cos {\gamma _{1,ua}}, \end{aligned}$$

where $\Upsilon = e^{-i \gamma _{1,uv} C_{uv}}e^{-i \sum _{a \in V_u\setminus {v}} \gamma _{1,ua} C_{ua}}$, and $\Upsilon ^\dag$ is its Hermitian conjugate. By symmetry, the term for $Z_uY_v$ is $-\sin {\gamma _{1,uv}}\prod _{b \in V_v\setminus {u}}\cos {\gamma _{1,vb}}$, where $V_v$ is the neighborhood of *v* in *V*. Factoring in the coefficient $-1/2$ of $Z_uZ_v$ in $C_{uv}$ gives the final two terms in the theorem.

Now, let us conjugate the last term of Eq. (3). Doing so, we get

$$\begin{aligned} {\left\langle s \right| }e^{i \sum _{gh \in E} \gamma _{1,gh} C_{gh}}Y_uY_v e^{-i \sum _{gh \in E} \gamma _{1,gh} C_{gh}} {\left| s \right\rangle } \\&\quad = {\left\langle s \right| }\prod _{a \in V_u\setminus v}({\mathbb {I}}\cos {\gamma _{1,ua}}- i\sin {\gamma _{1,ua}}Z_uZ_a)\times \prod _{b \in V_v\setminus u}({\mathbb {I}}\cos {\gamma _{1,vb}}-i\sin {\gamma _{1,vb}}Z_vZ_b)Y_uY_v{\left| s \right\rangle } \ \end{aligned}$$

The simplest terms that contribute to the expected value are of the form

$$\begin{aligned} \sin {\gamma _{1,uc}}\sin {\gamma _{1,vc}}\prod _{x \ne y}\cos {\gamma _{1,ux}}\cos {\gamma _{1,vy}} \end{aligned}$$

and there are *f* of these where *f* is the number of triangles containing *uv*. The higher order terms only contribute to the expected value if there are triangles in the graph. Thus, the last term of Eqn. (3) contributes nothing to the expected value of triangle-free graphs.

Combining these expressions gives the theorem. $\square$

### Star graphs

First, we will show that $\langle C_{ij} \rangle$ approaches 0.75 as *n* tends to infinity for QAOA. Since there are $n-1$ edges in a star on *n* vertices, this implies $\langle C \rangle$ tends to $0.75(n-1)$. Additionally, $n-1$ is the size of the optimal MaxCut solution, so $\langle C \rangle _1/C_\mathrm {max} = 0.75$.

Wang, Hadfield, Jiang, and Rieffel showed that^57^

4 $$\begin{aligned} \langle C_{ij} \rangle _1 = \frac{1}{2}+\frac{1}{4}(\sin {4\beta }\sin {\gamma })(\cos ^{d}{\gamma }+\cos ^{e}{\gamma }) -\frac{1}{4}(\sin ^2{2\beta }\cos ^{d+e-f}{\gamma })(1-\cos ^{f}{2\gamma }) \end{aligned}$$

where *d* is the $\deg (i)-1$, *e* is the $\deg (j)-1$ and *f* is the number of triangles containing edge *ij*^56,57^.

Let us consider the above formula applied to a star graph. Without loss of generality, let *j* be the center of each star. Then $d = 0$, $e = n-2$, and $f = 0$, since star graphs are trees. For each edge of the star, Eq. (4) reduces to

$$\begin{aligned} \langle C_{ij} \rangle _1 = \frac{1}{2}+\frac{1}{4}(\sin {4\beta }\sin {\gamma })(1+\cos ^{n-2}{\gamma }). \end{aligned}$$

We set $\beta = \pi /8$, which implies $\sin {4\beta } = 1$, since only one trigonometric function has $\beta$ as an argument. As *n* tends to infinity, note $\cos ^{n-2}{\gamma }$ tends to zero unless $\gamma = k\pi$ for some $k \in {\mathbf {N}}$. However, if $\gamma = k\pi$, $\sin {\gamma } =0$. Thus, this quantity is maximized when $\gamma \ne k\pi$, which implies $\langle C_{ij} \rangle _1$ tends to 0.75 for star graphs.

In order to prove $\langle C \rangle ^\mathrm {ma} = n-1$ for ma-QAOA on star graphs, we examine Theorem 4.1. Without loss of generality, let *u* be a leaf vertex and *v* be the center. Note that the first product is empty, since the leaf vertices have no neighbors except the center. Thus, Theorem 4.1 reduces to

$$\begin{aligned} {\left\langle \vec {\gamma _1}\vec {\beta _1} \right| } C_{uv} {\left| \vec {\gamma _1}\vec {\beta _1} \right\rangle } = \frac{1}{2}+ \frac{1}{2}\sin {\gamma _{1,uv}}(\cos {\beta _{1,v}'}\sin {\beta _{1,u}'}+\cos {\beta _{1,u}'}\sin {\beta _{1,v}'} \prod _{x}\cos {\gamma _{1,vx}}) \end{aligned}$$

Now, recall ${\left\langle \vec {\gamma _1}\vec {\beta _1} \right| } C_{uv} {\left| \vec {\gamma _1}\vec {\beta _1} \right\rangle } \le 1$, as two vertices that have an edge between them add one to the objective function if they are in different sets. In order to obtain equality, we can set $\gamma _{1,uv} = \pi /2$, as it is an argument for only a single sine term. Next, note that if either term in the parenthesis is one, the other must be zero. Also, setting one term equal to one allows gives an expected value of one for the edge. Let $\beta _{1,u}' = \pi /2$ and $\beta _{1,v}' = 0$. Then $\cos {\beta _{1,v}'} = \sin {\beta _{1,u}'}= 1$ while $\cos {\beta _{1,u}'} = \sin {\beta _{1,v}'}= 0$. Thus, the first term in the parenthesis is one and the second is zero. This allows us to set $\gamma _{1,vx} = \pi /2$ for all $x \in Nbhd(v)$. Since each of the $n-1$ edges in the star are described similarly, $\langle C \rangle _1^\mathrm {ma} = n-1$ for all *n*. The size of the optimal cut on a star graph is $n-1$, so $\langle C \rangle _1^\mathrm {ma}/C_\mathrm {max} = 1$.

### Setup for computational results

In order to calculate the angles that maximize $\langle C \rangle _p$ and $\langle C\rangle _1^\mathrm {ma}$ for the eight vertex graphs, we used the Broyden-Fletcher-Goldfarb-Shanno (BFGS) algorithm^58^. The algorithm inputs an initial collection of angles and then uses a numerical gradient and second order approximate Hessian to find angles that converge to local maxima of $\langle C \rangle _p$ and $\langle C\rangle _1^\mathrm {ma}$. For the eight vertex graphs, one-hundred random seeds were used to optimize $\langle C \rangle ^\mathrm {ma}_1$. The results for the $\langle C \rangle _p$ were taken from the online dataset^51^ of Ref.^35^, where we performed an exhaustive analysis of QAOA performance on small graphs. These used fifty seeds for $p=1$, one-hundred seeds for $p=2$, and one-thousand seeds for $p=3$.

For the fifty and one-hundred vertex graphs, we used the method of moving asymptotes (MMA) algorithm^59,60^, but note that calculations with BFGS gave similar results. The $\langle C\rangle _1$ were computed using Eq. (4) and the reported results were taken as the best from one-thousand initial seeds in MMA optimizations. The $\langle C\rangle _1^\mathrm {ma}$ were computed with Theorem 4.1 and MMA optimization. We report results as the best optimized values from one-thousand seeds at $n=50$ and from one-hundred seeds at $n=100$.

## Data Availability

The datasets generated during and/or analysed during the current study are available in the Multi-Angle-QAOA repository, https://code.ornl.gov/5ci/multi-angle-qaoa.
